# Glenohumeral Joint Preservation: A Review of Management Options for Young, Active Patients with Osteoarthritis

**DOI:** 10.1155/2012/160923

**Published:** 2012-03-27

**Authors:** Olivier A. van der Meijden, Trevor R. Gaskill, Peter J. Millett

**Affiliations:** The Steadman Clinic, Steadman Philippon Research Institute, 181 West Meadow Drive, Suite 400, Vail, CO 81657, USA

## Abstract

The management of osteoarthritis of the shoulder in young, active patients is a challenge, and the optimal treatment has yet to be completely established. Many of these patients wish to maintain a high level of activity, and arthroplasty may not be a practical treatment option. It is these patients who may be excellent candidates for joint-preservation procedures in an effort to avoid or delay joint replacement. Several palliative and restorative techniques are currently optional. Joint debridement has shown good results and a combination of arthroscopic debridement with a capsular release, humeral osteoplasty, and transcapsular axillary nerve decompression seems promising when humeral osteophytes are present. Currently, microfracture seems the most studied reparative treatment modality available. Other techniques, such as autologous chondrocyte implantation and osteochondral transfers, have reportedly shown potential but are currently mainly still investigational procedures. This paper gives an overview of the currently available joint preserving surgical techniques for glenohumeral osteoarthritis.

## 1. Introduction

**Figure 1:**
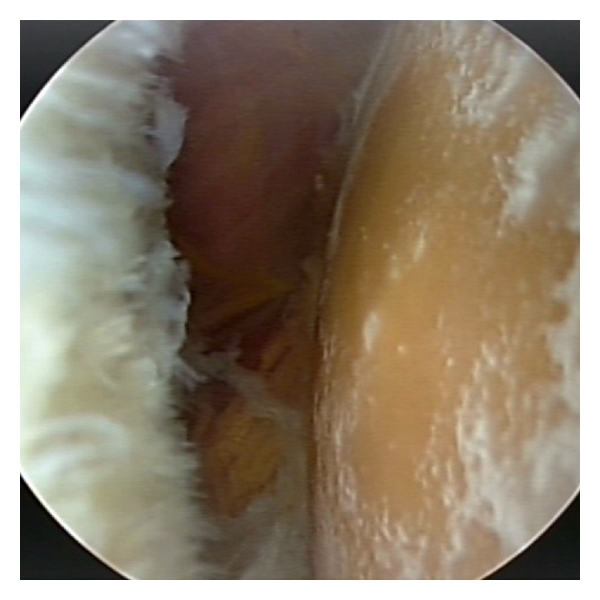
Arthroscopic view of severe osteoarthritis of the right humeral head in a 53-year-old female.

Osteoarthritis (OA) is the most frequent cause of disability in the USA [1]. It is suggested that as many as 50 million adults suffer from this gradual, progressive joint failure [2]. The prevalence of OA increases with age, typically manifesting after the sixth decade of life, and women appear to be more susceptible than men [2]. Though less prevalent than OA of the knee and hip, OA of the shoulder (Figure 1) can be equally debilitating [3].

Treatment of shoulder OA is typically based on the patient's age, severity of symptoms, level of activity, radiographic findings, and medical co morbidities. Nonoperative treatment options include activity modification, physical therapy, oral anti-inflammatories, and intra-articular injections, each exhibiting varying reported efficacy rates [4]. If conservative options fail, surgical treatment should be considered. As in other joints affected by severe OA, the most definitive treatment modality is joint arthroplasty. Specifically, shoulder arthroplasty reliably results in pain reduction and functional improvement but has been primarily studied in older arthritic patients with lower functional demands [5–11].

The management of shoulder OA in young active patients remains a challenge and the optimal treatment has yet to be completely established [12]. Many young and active patients with early stage joint degeneration wish to maintain a high level of activity because of recreational interests or occupational demands. In these cases, arthroplasty may not be a practical treatment option secondary to concerns regarding implant durability [13]. It is these patients who may be excellent candidates for joint-preservation procedures in an effort to avoid or delay joint replacement.

Glenohumeral joint preservation is not a novel concept. Previous authors have described arthroscopic debridement and capsular release [14–16], microfracture [17–19], corrective osteotomies, osteochondral transfers, and chondral implantations [20, 21] with satisfactory results. More recently arthroscopic debridement and capsular release has been coupled with humeral osteoplasty and axillary nerve decompression in an effort to improve reported outcomes [22]. These procedures also typically have the benefit of less surgical morbidity and a quicker postoperative recovery.

The long-term outcomes of glenohumeral preservation techniques are presently unknown but clinical experience has shown that many patients do well with these procedures and delay the need for prosthetic shoulder arthroplasty. Early results from published studies do indicate that satisfactory short-term outcomes can be expected, but these procedures have yet to show that they can halt the arthritic progression [14–16]. They may, however, provide a window of improved function for this young and active population. The purpose of this paper is to give an overview of the currently available joint preserving surgical techniques and report on the evidence supporting procedures available for the young and active patient population with shoulder OA.

## 2. Articular Cartilage

Like other diarthrodial joints, the glenohumeral articular surfaces are similarly covered with hyaline cartilage which on the glenoid side is thicker at the periphery than centrally. By contrast, the humeral articular cartilage thickness is exactly the opposite; the cartilage thickness at the periphery is approximately 1 mm, increasing from 1.2 to 1.3 mm at the center of the humeral head [23]. It is thought that this variation in cartilage thickness may increase the congruency of the joint's osseous structures [24].

The etiology of articular cartilage injury includes trauma, iatrogenic causes, instability, avascular necrosis, osteochondritis dissecans, chemically induced, and osteoarthritis [25]. Cartilage defects rarely heal spontaneously and generally require surgical intervention because of the poor vascularization of articular cartilage and the presence of few undifferentiated cell populations able to respond to degenerative or traumatic injury [26].

**Figure 2:**
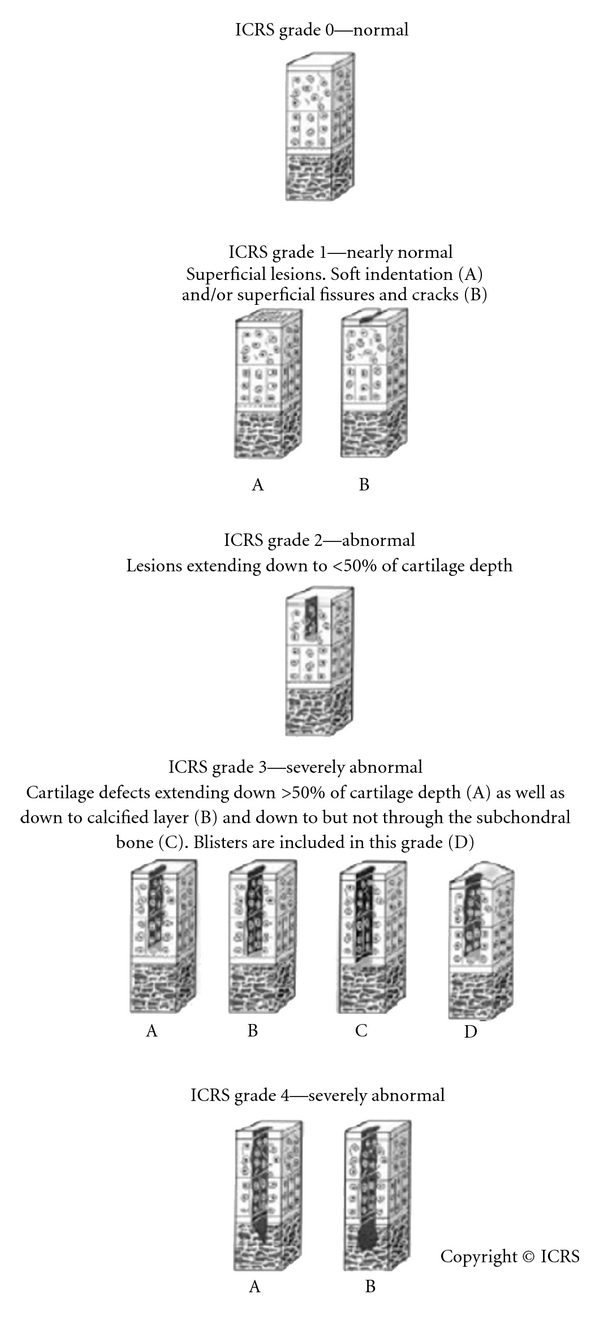
The International Cartilage Repair Society (ICRS) Cartilage Lesion Classification System. Reprinted with permission from the ICRS Cartilage Injury Evaluation Package (http://www.cartilage.org/).

Osteoarthritis affects not only the articular cartilage but also the bone and the capsule of the joint. This results in osteophyte formation, subchondral sclerosis, and capsular thickening. Often these manifestations are as important to treat as the chondral damage present. Lesions of the cartilage have been historically graded according to the Outerbridge classification grade I–IV [27]. In 2003, the International Cartilage Repair Society (ICRS) published the ICRS Hyaline Cartilage Lesion Classification System, a modification of the Outerbridge classification, which is currently used as the international standard [28] (Figure 2). 

Focal lesions of articular cartilage of the glenohumeral joint are often encountered during arthroscopic treatment of other shoulder injuries [29–33]. Several authors report grade III to IV chondral lesions in 5%–17% of patients undergoing rotator cuff repair [30, 31], and 6% to 29% of those being treated for impingement symptoms [29, 32]. Unpublished data from our clinical database confirms these previous reports, revealing a 12.4% prevalence of high-grade cartilage lesions in over 2000 patients who have undergone arthroscopic surgery [33]. It is critical to realize that joint preservation techniques should not only be employed once arthrosis occurs, but also as an important adjunct to other procedures in an effort to minimize damage and slow progression to arthritis.

## 3. Current Treatment Options

### 3.1. Nonoperative Treatment

Though nonoperative management of glenohumeral OA will not ultimately alter the progression of disease, it can be effective in mitigating symptoms. Activity modification is often an initial step in this process. Although the recently published treatment guidelines of the American Academy of Orthopaedic Surgeons are unable to recommend for or against physical therapy, manual therapy, or manipulation based on the available published literature [4], these modalities are often prescribed because they represent minimal risk to the patient, and there certainly have been anecdotal reports of success. The same guidelines were also unable to recommend for or against the use of intra-articular corticosteroid injections or oral pharmacotherapy. Anecdotal experience again suggests that these treatments are effective for many patients, but often only for a limited duration of time. Because these therapies also pose minimal patient risk, they are often initiated prior to surgical intervention. Randomized trials do exist indicating that nonsteroidal anti-inflammatory drugs (NSAIDs) are more effective than both paracetamol and placebo for pain relief of arthritic conditions [34, 35]. It is important, however, to be aware of the increased risk of gastro-intestinal and cardiovascular side effects when considering NSAID prescription for this cause [35]. Some evidence also exists supporting glenohumeral viscosupplementation for glenohumeral arthrosis. Silverstein et al. reported that glenohumeral viscosupplementation resulted in a significant improvement in shoulder pain and function outcome scores 6 months following injection [36].

### 3.2. Operative Treatment: Symptom Relief

#### 3.2.1. Debridement with Capsular Release

Arthroscopic debridement of the shoulder with glenohumeral arthrosis has been used to treat patients with (early) OA of the shoulder. By stabilizing cartilage lesions, eliminating mechanical symptoms, and releasing capsular contractures, satisfactory outcomes have been obtained in small cohorts of patients as reported by several authors [14, 15, 37]. Weinstein et al. described good results from arthroscopic debridement alone in patients with mild or minimal arthritic change and less favorable results in patients with more advanced changes (average age 46, range 27–72 years old) [37]. Richards et al. combined arthroscopic debridement with capsular release in young patients (mean age 56 ± 12 years). This resulted in improved glenohumeral motion and an average symptom free period of 9 months in a small cohort of patients [15]. More recently, Van Thiel et al. described a significant decrease in pain in 55 of 71 patients, mean age 47 years old (range 18–77), after arthroscopic glenohumeral debridement at a mean of 27 months postoperatively [14]. Although arthroscopic intervention is not likely to halt arthritic progression, it may provide a period of improved pain and function, thereby delaying a larger operation in those with physically demanding occupations or recreational interests.

In some circumstances arthrosis of the glenohumeral joint is accompanied by large inferior humeral osteophytes. Previous authors have suggested that arthroscopic debridement procedures are less efficacious when osteophytes are present [37]. It has also been shown in cadaveric studies that the axillary nerve runs in close proximity to the inferior glenohumeral capsule [38, 39]. Therefore, it may be possible for a humeral osteophyte of sufficient size to compress the axillary nerve and potentially result in posterior or lateral shoulder pain (axillary nerve distribution) similar to that experienced with quadrilateral space syndrome. This may partially explain less favorable results reported in this subset of patients if the axillary nerve is not properly decompressed.

**Figure 3:**
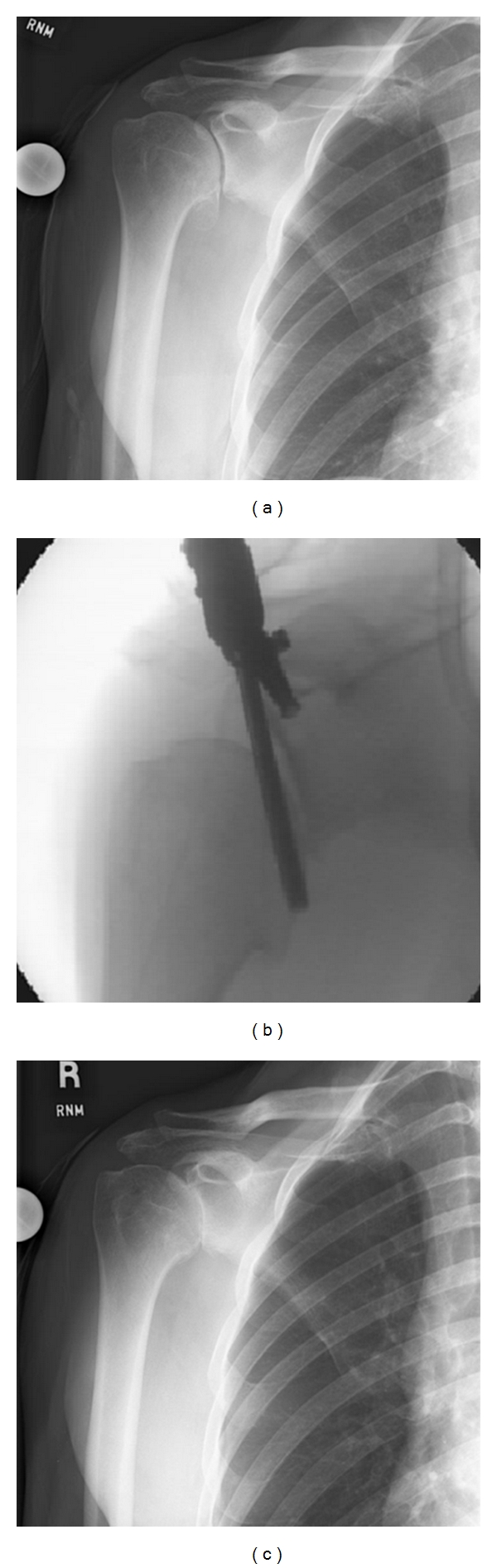
Preoperative (a) Intraoperative (b), and postoperative (c) radiologic imaging of the Comprehensive Arthroscopic Management procedure of an osteoarthritic shoulder of a 52-year-old male. (b) shows the osteoplasty of the inferior humeral osteophyte with the arthroscopic burr.

Therefore, we have recently begun combining typical arthroscopic debridement and capsular release, with a meticulous humeral osteoplasty and an arthroscopic trans-capsular axillary nerve decompression (CAM; Comprehensive Arthroscopic Management) [22]. In our recently published study, we reported on the results of 28 procedures in 27 patients [40]. All had severe glenohumeral osteoarthritis and otherwise would have been candidates for total shoulder arthroplasty. At an average of 20 months postoperatively, we had a high patient satisfaction rate, decreased pain, improved range of motion, and improved ASES scores. We had good survivorship as well; only one patient had persistent pain that was severe enough that he elected to undergo total shoulder arthroplasty. Younger patients or those who place considerable functional demands on the glenohumeral joint may be excellent candidates for this type of procedure. Several of the patients in this series are now many years out from their CAM procedure and are still satisfied and highly functional. For the appropriate patient, the addition of an axillary nerve decompression and humeral osteoplasty may provide symptomatic relief that is greater than simple debridement alone (Figures 3(a)–3(c)) [40]. These data suggest that the CAM procedure may be a promising alternative for young and active patients with early shoulder OA. In the future this procedure may be able to be combined with other restorative types of procedures, but further research is required.

### 3.3. Operative Treatment: Regeneration, Repair, or Reconstruction

#### 3.3.1. Microfracture

**Figure 4:**
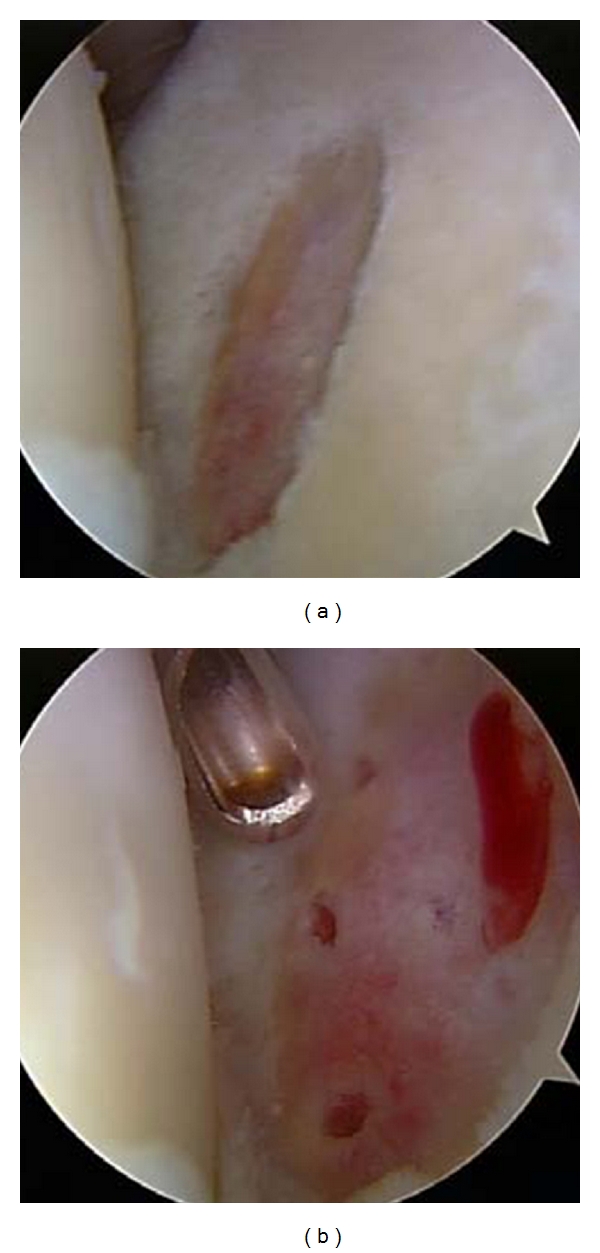
Arthroscopic images of preparation for (a) and result of (b) microfracture technique of a 2 × 2 cm glenoid lesion in a 65-year-old male.

The technique of microfracture as a marrow-stimulation procedure was first described as a repair option for full-thickness focal cartilage defect in the knee with good clinical outcomes and low surgical morbidity [41, 42]. Recently, this technique has also been increasingly described successfully for focal defects of the glenohumeral joint [17–19]. Millett et al. reported significant improvement at an average follow-up of 47 months in pain scores and functional outcome in 81% of 31 cases in a population of grade IV cartilage lesions (average age 43, range 19–59 years old). The greatest improvements were noted in small lesions of the humerus and the worst in patients with both humeral and glenoid defects [17]. Frank et al. recently reported results of 17 cases at an average follow-up 27.8 months (average age 37, range 18–35 years old). They also reported significant improvements in pain and functional outcomes scores. The average size of treated lesions was 5.07 cm² for humeral lesions and 1.66 cm² for glenoid defects [18]. Finally, a small study by Siebold et al. involving 5 patients with a mean age of 32 and an age range of 16–56 years old also showed significant improvements in pain and functional outcomes scores after performing a combination of microfracture and periosteal flap for focal chondral lesions (Figures 4(a)-4(b)) [19].

#### 3.3.2. Autologous Osteochondral Transfers

Similar to microfracture, the technique of autologous osteochondral transfer has been extensively studied and appears to be effective for treatment of full-thickness cartilage defects of the knee [43, 44]. While microfracture is performed arthroscopically, chondral transfers often require an open procedure and also harbors the risk of donor site morbidity. By contrast osteochondral transfers provide the advantage of facilitating transfer of both cartilage and bone. Therefore it is capable of treating bone defects in addition to full-thickness cartilage lesions. Little is presently known regarding the results of this procedure in the glenohumeral joint. Scheibel et al. presented a small case series of 8 grade IV lesions with 32 months of follow-up; the average age of included patients was 43 years old (range 23–57) [20]. Autograft osteochondral transfers from the knee were performed, resulting in improved shoulder outcome scores; however donor site morbidity of the knee was reported to be 20%.

#### 3.3.3. Autologous Chondrocyte Implantation (ACI)

ACI techniques have also been successful for the treatment of cartilage lesions of the knee [45]. ACI provides the advantage of eliminating the risk of donor site morbidity, though it must be performed as a staged approach using an open surgical technique. Young and active patients with high demand of shoulder function and isolated focal lesions of the humeral cartilage seem most fit for this type of treatment. To date, only one case report of a young baseball player has been published reporting good results 12 months after surgery [21].

#### 3.3.4. Osteochondral Allografts

**Figure 5:**
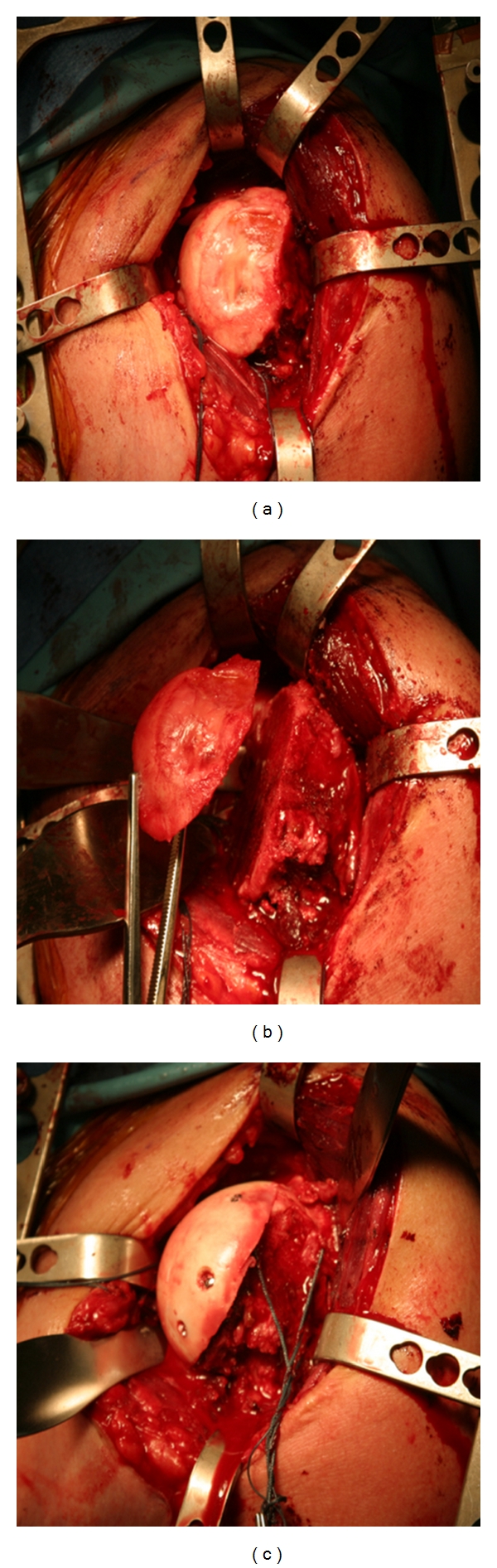
Large humeral head cartilage defect (a), excised (b) and replaced with an osteochondral allograft (c) in a patient younger than 50 years old.

 Osteochondral Allografts can be used for large full-thickness cartilage lesions of the humerus and also provide the advantage of avoiding donor-site morbidity risk (Figures 5(a)–5(c)). However, possible disadvantages include limited chondrocyte viability, loss of matrix structure, and transmission of disease. As a restorative technique, allograft transfer has proven efficacious in other joints [46–48] but their application to the glenohumeral joint is relatively uncommon. The most frequent use of allograft in the glenohumeral joint is for the treatment of engaging Hill-Sachs lesions and bony deficits resulting from glenohumeral instability following tumor resections [49, 50]. Recently Krishnan et al. published promising early results in 4 patients, mean age 47, of an all-arthroscopic technique for osteochondral allograft resurfacing of both the glenoid and humeral articular surface [51]. Though possibly a promising alternative for young and active patients with early shoulder OA, further research and follow-up is required.

#### 3.3.5. Biologic Resurfacing/Interposition Arthroplasty

Though still primarily investigational, treatment of focal chondral defects of the glenoid using biologic glenoid resurfacing was first described in the late 1980s [52]. Since then several different techniques have been described [53, 54].

Nicholson et al. conducted a prospective study in which 36 patients, average age 51 and range 30–75 years old, were treated with humeral hemiarthroplasty combined with anterior capsular interposition in 7 shoulders, autogenous fascia lata in 11 patients, and Achilles tendon allograft in the remaining 18 shoulders [52]. The authors report pain relief comparable to those of total shoulder arthroplasty. Ball et al. reported similar favorable results at two-year follow-up after treating 6 patients, average age 48 years old (range 33–54), with fascia lata and anterior capsule glenoid resurfacing techniques [55].

Other authors reported on open [56] and later arthroscopic [57] glenoid resurfacing techniques using lateral meniscus allografts. A cadaveric study by Pennington and Bartz showed that lateral meniscus allograft significantly reduced contact forces as compared to the medial meniscus [56]. Huijsmans et al. reported overall good results with a considerable complication rate using lateral meniscus allografts to resurface 30 glenoids of young patients (mean age 42 and range 18–52) with glenohumeral OA in combination with humeral hemiarthroplasty [53].

Elhassan et al. described an arthroscopic technique of glenoid resurfacing using a GraftJacket (Wright Medical Technology, Arlington, TN, USA), consisting of processed human donor skin [58]. Promising preliminary results using GraftJacket interposition have been reported at 6-months follow-up in 6 patients [54].

**Figure 6:**
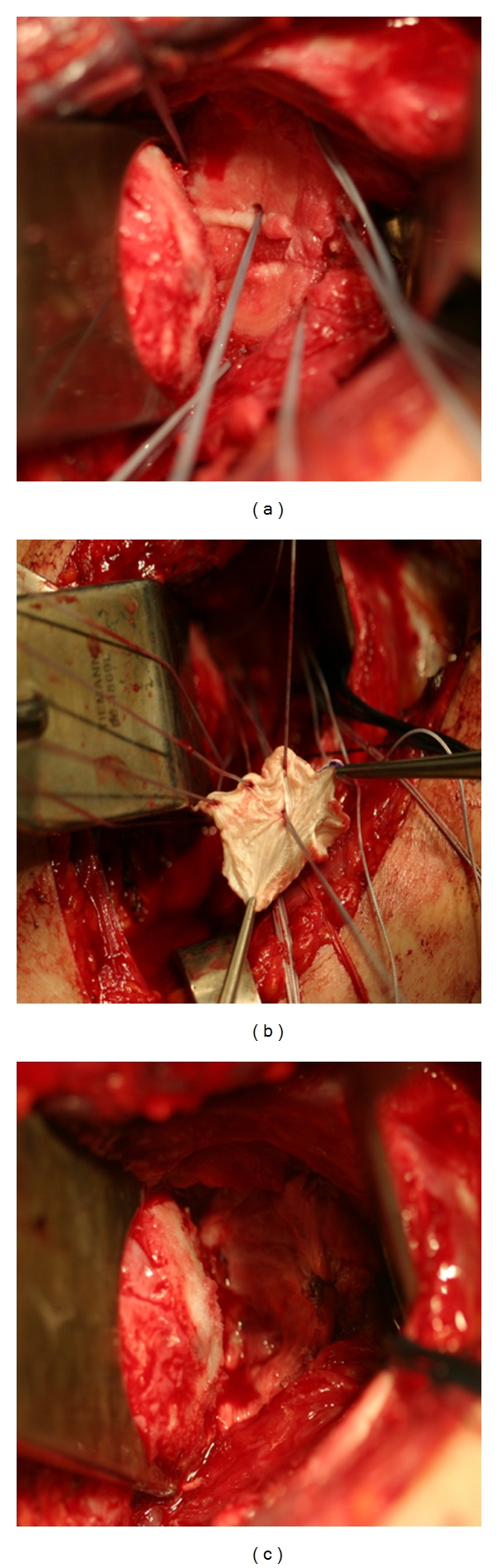
Preparation of the glenoid (a) for soft-tissue (b) resurfacing of the glenoid in a patient younger than 50 years old with a glenoid cartilage defect (c).

Though many of these techniques have shown some promising early results in terms of pain relief, most recently Gobezie et al. published devastating results of 13 patients, mean age 34 and range 18–49, treated with soft-tissue resurfacing of the glenoid alongside arthroplasty of the humeral head [59]. Revision total shoulder arthroplasty was required by 77% of patients at a mean of 14 months postoperatively because of persistent pain and decreased range of motion. The authors concluded that this procedure is not a reliable method of treatment in young patients with shoulder OA based on both the poor clinical outcome and absence of the graft acting as a durable glenoid surface (Figures 6(a)–6(c)).

## 4. Conclusions

The management of osteochondral pathology of the shoulder in young active patients is a challenge, and the optimal treatment has yet to be completely established. If nonoperative treatment fails, several restorative and palliative surgical techniques are currently optional. Historically, joint debridement has shown good results, and a combination of arthroscopic debridement with a capsular release, humeral osteoplasty, and transcapsular axillary nerve decompression seems to be a promising procedural advance, particularly when large humeral osteophytes are present.

Currently, microfracture seems the most effective reparative treatment modality available for focal cartilage lesions. Though several other techniques have been described, such as autologous chondrocyte implantation, autologous osteochondral transfers, osteochondral allografts, and biologic soft tissue interposition arthroplasties, they are currently mainly still investigational procedures. Long-term results and documentation of the natural history of disease following these procedures are required to optimize joint-preservation treatment.
